# Rv0100: An essential acyl carrier protein from *M*. *tuberculosis* important in dormancy

**DOI:** 10.1371/journal.pone.0304876

**Published:** 2024-06-07

**Authors:** Hiten J. Gutka, Jasper Marc G. Bondoc, Ryan Patwell, Shahebraj Khan, Edyta M. Grzelak, Rajendra Goswami, Martin I. Voskuil, Farahnaz Movahedzadeh

**Affiliations:** 1 Institute for Tuberculosis Research, College of Pharmacy, University of Illinois at Chicago, Chicago, Illinois, United States of America; 2 Department of Neuropeptide Research, Central Institute for Mental Health, Mannheim, Germany; 3 Department of Microbiology, School of Medicine, University of Colorado Denver, Aurora, Colorado, United States of America; 4 Department of Pharmaceutical Sciences, College of Pharmacy, University of Illinois at Chicago, Chicago, Illinois, United States of America; Rutgers Biomedical and Health Sciences, UNITED STATES

## Abstract

We have identified an acyl-carrier protein, Rv0100, that is up-regulated in a dormancy model. This protein plays a critical role in the fatty acid biosynthesis pathway, which is important for energy storage and cell wall synthesis in *Mycobacterium tuberculosis* (MTB). Knocking out the *Rv0100* gene resulted in a significant reduction of growth compared to wild-type MTB in the Wayne model of non-replicating persistence. We have also shown that *Rv0100* is essential for the growth and survival of this pathogen during infection in mice and a macrophage model. Furthermore, knocking out *Rv0100* disrupted the synthesis of phthiocerol dimycocerosates, the virulence-enhancing lipids produced by MTB and *Mycobacterium bovis*. We hypothesize that this essential gene contributes to MTB virulence in the state of latent infection. Therefore, inhibitors targeting this gene could prove to be potent antibacterial agents against this pathogen.

## Introduction

*Mycobacterium tuberculosis* (MTB), a causative agent of tuberculosis (TB), has infected more than a quarter of the human population and was the second leading cause of death by a single infectious agent in 2022 [1]. The infection usually develops into an asymptomatic state of latency which may last for many years. Changes in environmental conditions, such as compromised immunity of the host, may induce the dormant bacterium to begin proliferating and cause a shift from latent infection to pathogenesis. Furthermore, the increasing prevalence of drug resistance to the antibiotics that are part of the first-line treatment regimen has led to the proliferation of multiple drug-resistant (MDR) TB and extensively drug-resistant (XDR) TB [2, 3]. Consequently, there is an urgent need to identify new targets and more effective anti-mycobacterial agents to combat this disease.

Sequencing the MTB genome has revealed that over one third of its genome consists of unannotated genes; 15% with an unknown function and 23% associated with only hypothetical proteins. Subsequent proteomics and microarray analyses found many of these unannotated genes showed significant changes in expression under hypoxic conditions [4–9]. A basic understanding of the metabolic and regulatory pathways that allow MTB to maintain dormancy during a latent infection will be crucial to future therapeutic development. Yet, we still lack enough knowledge of these pathways to adequately describe the mechanisms of dormancy in MTB. Additionally, drug discovery efforts will be better served by focusing on proteins from MTB that do not share any functional homology with the human host, such as the unannotated protein Rv0100.

Rv0100 is a small, soluble protein that is composed of 78 amino acids with 2 cysteines and is likely located in the cytoplasm of MTB. Rv0100 was previously suggested to be essential for MTB survival in a mouse model of TB infection. This study by Sassetti and Rubin suggested that approximately 5% of the MTB genome was critical for growth, including *Rv0100* [10].

**Fig 1:**
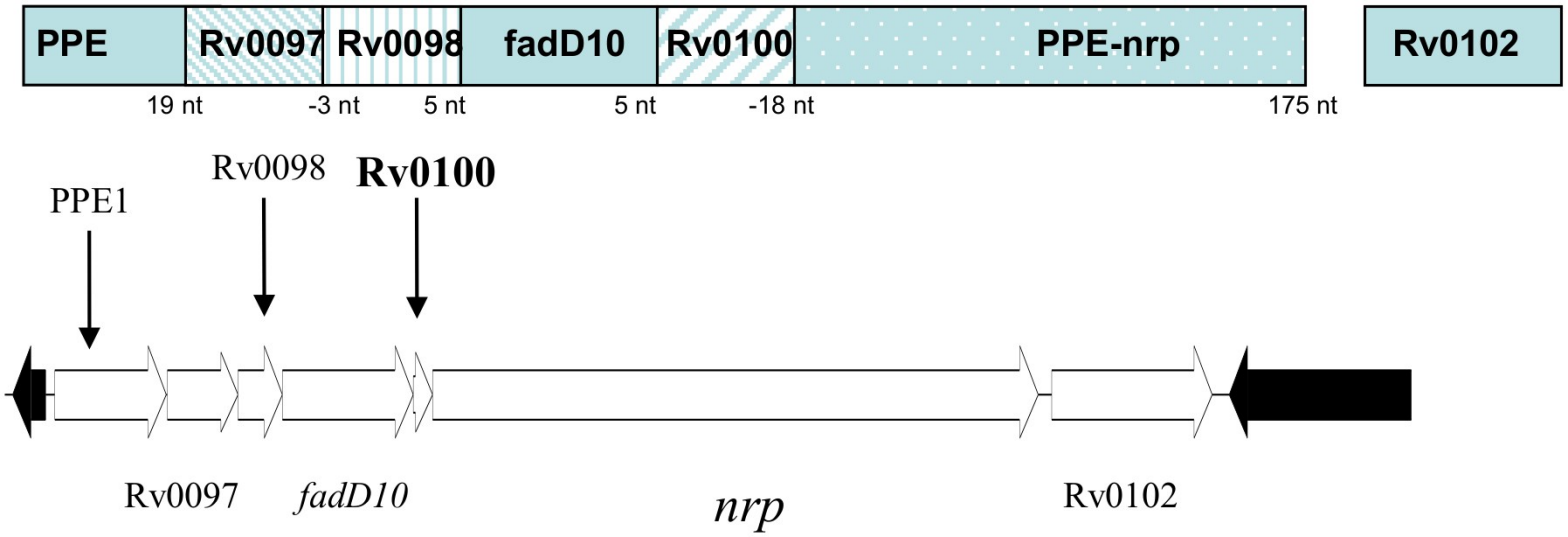
Rv0100 operon. The genomic structure surrounding *Rv0100* including all the genes contained in the same operon. This includes all genes contained within the same operon as our target of interest, *Rv0100*. Here, the nrp gene (encoding the Rv0101 protein) is shown to be only 18 nucleotides apart from *Rv0100*. The overlapping stop codon of *Rv0100* and start codon of *Rv0101* make them likely protein partners.

Rv0100 is predicted to be an acyl carrier protein (ACP) with a phosphopantetheine attachment site [19], suggesting it plays a critical role in the fatty acid biosynthesis pathway, which is one of the primary targets for anti-mycobacterial therapeutics. Fatty acid precursors are important for cell wall synthesis and the energy storage required to maintain dormancy. In particular, MTB uses triacylglycerol to accumulate lipid droplets to initiate a dormancy-like phenotype [11]. *Rv0100* is contained within an operon (Fig 1) that includes the nrp gene (*Rv0101*), which encodes an adenosine monophosphate-dependent peptide synthase involved in either polyketide non-ribosomal peptide synthase or fatty acid degradation. The operon also encodes a protein predicted to be a fatty acid CoA ligase (FadD10, *Rv0099*). Both *Rv0100* and *Rv0101* have phosphopantetheine attachment sites. Fatty acid adenylating enzymes (FADD) and fatty acyl-AMP ligase (FAAL) are essential for mycobacteria, specifically for linking fatty acids and polyketide synthesis [12, 13]. FAAL has been shown to be responsible for transferring fatty acids to Rv0100 [14]. When MTB transitions into a hypoxic stage, the energy supply pathway may shift to utilize fatty acids as an energy source, and *Rv0100* and surrounding genes in the operon may be involved in this process. The entire operon is conserved in the pathogens *Mycobacterium bovis* (M. bovis), *Mycobacterium leprae* and *Mycobacterium avium*. Based on bioinformatics methods to predict protein partners, *Rv0100* is co-expressed with *Rv0101*, as the *Rv0100* stop codon overlaps with the start codon of *Rv0101*, with the genes being 18 nucleotides apart on the genome. Rv0100 may also be a protein partner with FadD10, as these genes are only 5 nucleotides apart. Additionally, this highly conserved group of genes has been implicated in the biosynthesis of phthiocerol dimycocerosates (PDIMs), the virulence-enhancing lipids produced by MTB and M. bovis [15]. Indeed, Hotter et. al (2005) found that the mutation of the *Mb0100* gene (equivalent to MTB *Rv0097*) disrupted the synthesis of PDIMs and caused a loss of virulence.

Genomic sequencing studies as well as protein analyses of a persistent MTB mutant have shown that the formation of the mutant persistence phenotype is related to several pathways involving lipid biosynthesis and other carbon pathways [10, 16]. In particular, under hypoxic conditions, lipid inclusion bodies play an important role in drug resistance [17]. Further, several studies have shown that statins (inhibitors of cholesterol biosynthesis) as well as other acyl protein metabolizing enzymes have anti-tubercular effects [18], which suggests that acyl carrying proteins or other key elements of the lipid metabolism pathway can serve as promising drug targets.

In addition to Rv0100, there are other ACPs identified in the MTB genome such as Rv2244, Rv0033, and Rv1344 [19]. These three proteins share an identity of 15–23% and a similarity of 26–39% with Rv0100 [20]. However, these proteins were not found to be up-regulated in the whole genome expression experiments mentioned above while, in contrast, *Rv0100* has been shown to be upregulated in several other studies using a dormancy model under hypoxic conditions [6, 21, 22]. Therefore, the other three ACPs are less likely to be major contributors to survival during a state of latent infection.

Our lab has previously overexpressed and purified the Rv0100 protein [23, 24]. However, understanding the function of *Rv0100* in a mouse model of TB infection and in a dormancy-like state will be necessary to determine its potential as a drug target. In this study, we generated *Rv0100* knockout mutant (KO) and a complement strain to determine if *Rv0100* is essential for the growth and survival of MTB in infected mice, the Wayne model of persistence and a macrophage model.

## Materials and methods

### Bacterial strains, plasmids and media

Bacterial strains and plasmids used are shown in Table 1. *E*. *coli* DH5α was used for all plasmid constructions. MTB H37Rv was cultured on Middlebrook 7H10 agar with 10% oleic acid-albumin-dextrose-catalase (OADC) supplement. Middlebrook 7H9 broth with 10% OADC supplement and 0.05% Tween 80, Sauton’s medium (0.025% Tyloxypol, 2% glycerol) [25] or Dubos Tween-albumin were used to grow liquid cultures. Hygromycin (100μg/ml), kanamycin (20μg/ml), gentamicin (10μg/ml) was added where appropriate.

**Table 1:** Strains and plasmids used for the generation of *Rv0100* KO and complement strains.

Strains/plasmids	Characteristics	Source
*E*. *coli* DH5α	Common commercially available laboratory strain. Defined by the three primary mutations: ΔrecA1, ΔendA1, lacZΔM15	Invitrogen
*M*. *tuberculosis* H37Rv	Wild-type laboratory strain	ATCC 25618
FAME 122	*M*. *tuberculosis*::pFM220 (*Rv0100* SCO)	This study
pUC19	Encodes the N-terminal fragment of b-galactosidase (lacZa), (used for colony selection)	[26]
p2NIL	Gene manipulation vector, kan	[27]
pGOAL19	*hyg pAg*_*85*_*-lacZ sacB Pac*I cassette vector	[27]
pUC-Gm-int	pUC-based plasmid with HindIII cassette carrying gm and L5 int	[28]
pFM214	*Rv0100*+flanking::pUC19	This study
pFM216	pFM214::Δ*Rv0100*	This study
pFM218	p2NIL::*Rv0100*	This study
pFM220	pFM218 with *Pac*I cassette of pGOAL19 (delivery construct)	This study
pFM225	*Rv0100*:: pFM209	This study
pFM227	pFM225::HindIII cassette of pUC-Gm-int	This study

### PCR amplification and cloning of Rv0100

Cloning of Rv0100 was performed using standard techniques previously described in detail [27]. Briefly, MTB sequences of the target gene were retrieved from Genebank (NC_000962.2) and used to design primers for DNA amplification (Table 2). The primers, Rv0100 Flank-1 and Rv0100 Flank-2, were designed to amplify the target gene and 1kb of the upstream and downstream flanking region. The amplification reaction was performed with Platinum PCR Supermix High Fidelity (Invitrogen) according to the manufacturers protocol with an annealing temperature of 55°C. PCR products were then cloned into pUC19 generating the construct pFM214.

**Table 2:** List of primers used for the generation of the mutant *Rv0100* KO strain.

Rv0100 Flank-1: GGATCAGCTGGTCGGCGAAACCACCTACT
Rv0100 Flank-2: GGATCAGCTGGGTAATGCTCCTCACGAAGC
Rv0100 Inverse-1: GAGATCCGCCTCGTCGATATA
Rv0100 Inverse-2: CTGGAGGCTGTGTGCACC

### Inverse PCR reaction

To create a deletion in *Rv0100*, inverse PCR was first performed on pFM214 [27] with the primers Rv0100 Inverse-1 and Rv0100 Inverse-2 (Table 2) using Phusion High-Fidelity DNA Polymerase (NEB) following the manufacturers protocol. These primers are designed in such a way that the flanking regions and plasmid sequence of pFM214 are amplified leaving out most of the target gene’s coding sequence. Next, the amplified template was digested with DpnI for 1h at 37°C followed by blunt end religation to restore the circular form of the plasmid. The new plasmid, pFM216, contains the deletion in *Rv0100*. The deletion in the resulting plasmid was confirmed with sequencing.

### Cloning in p2NIL and cloning of the marker gene cassette

The SmaI restriction site present in pUC19 was used to digest the deleted versions of the target gene from the pFM216 plasmid and clone it into p2NIL. The pFM216 plasmid were digested with KpnI and BamHI and cloned into the KpnI and BamHI sites of p2NIL, generating the plasmid pFM218. A marker gene cassette derived from pGOAL19 was cloned into the PacI site of p218, generating the suicide delivery plasmid pFM220. The marker gene cassette contains the hygromycin resistance gene, the *lacZ* gene for blue/white selection, and the *sacB* gene that confers sucrose sensitivity. This set of genes is used for the selection of a single cross-over and ultimately a double cross-over.

In order to complement the *Rv0100* mutant, this gene was cloned into pFM209 [29] under the *Ag85* promoter, to produce pFM225. Then the HindIII cassette of pUC-Gm-int, which has the integration part and gentamicin gene, was cloned into the HindIII site of pFM225 to produce pFM227.

### In vitro growth under hypoxic conditions using the Wayne model

The effect of *Rv0100* deletion on growth under hypoxic conditions was performed using the commonly used protocol originally established by Wayne and Hayes [30]. Briefly, cultures of WT (H37Rv) MTB, along with the *Rv0100* KO mutant and the *Rv0100* complement strains were grown in Dubos-Tween-Albumin (Becton Dickinson) to an OD600 of 0.4 to 1. For hypoxic cultures, 16x100mm glass culture tubes containing 9ml DTA were inoculated to an OD580 of 0.04 and incubated at 37°C with 12mm magnetic stirrer bars stirring at 120rpm for up to two weeks. Screw-capped tubes were tightened to ensure an air-tight seal. Methylene blue 1.5ug/ml (final concentration) was added to one culture per strain as an indicator of oxygen depletion, which occurred completely by the 8^th^ day. Two tubes were sampled at each time point of 4, 7, and 14 days in addition to the day 0 control. Colony forming units (CFU) were counted by serial dilution and plated on Middlebrook 7H11 medium containing OADC after 4 weeks incubation at 37°C. CFU measurements were obtained by averaging two technical replicates each from two biological replicates per strain at days 7 and 14. Only one biological replicate and two technical replicates were prepared for day 0 readings.

### Detection and analysis of PDIMs

Given the involvement of Rv0100 in the fatty acid biosynthesis pathway, we wanted to know if its deletion would affect the synthesis of PDIMs, which are lipid components of the MTB cell wall that are known to be essential for its survival and virulence [31]. It is worth noting that while we observe no attenuation of growth compared to WT MTB when the *Rv0100* KO mutant is cultured in 7H9 + OADC media, our initial attempts to culture this mutant in Sauton’s media failed. However, we were able to achieve marginal growth by lowering the concentration of glycerol from 6% to 2% (S1 Fig) which allowed us to collect the pellicles necessary for the PDIM analysis described below.

Bacterial pellicles were collected from 200ml cultures after 20 days by pouring off the excess media. The bacteria were inactivated by heating at 95°C for 2h and then transferred to a solution of CHCl_3_/CH_3_OH (2:1, v/v) for incubation at room temperature overnight. The lipids were extracted twice with CHCl_3_/CH_3_OH (1:1, v/v), concentrated under vacuum, then washed three times with water and dried. This crude lipid extract contains phosphoglucoisomerase (PGI) and various members of the DIM family such as dimycocerosates of phthiocerol A (DIM A) and dimycocerosates of phthiodiolone (DIM B) [32]. Thin-layer chromatography (TLC) was performed as previously described [33]. Briefly, TLC was conducted on silica gel G60 plates (20x3x20cm; Merck) using petroleum ether/diethyl ether (9:1 v/v) as the eluent. Lipid compounds were visualized by spraying the plates with 10% phosphomolybdic acid in ethanol and heating. Two biological and two technical replicates were run for each gel across two experimental runs. It should be noted that heat inactivation of the bacterial cultures may have led to some loss of PDIMs however, the reported qualitative differences are relative to the WT MTB strain, which was treated identically to the other strains.

### In vivo growth and survival in mice

BALB/c female mice (n = 36) approximately 7–8 weeks old with an average weight of 20g were used for evaluating the effect of *Rv0100* gene knockout on MTB infection in vivo. The mice were randomly divided into three groups (n = 18/group) and infected via aerosol using a Glas-Col inhalation exposure system with 2x10^7^ CFU/ml of either WT, *Rv0100* KO or the *Rv0100* complement strain. Six mice from each group were sacrificed at post-infection days 1, 29 and 99. Carbon dioxide from a bottled source was used for asphyxiation followed by cervical dislocation. Whole lungs from individual mice were aseptically removed and homogenized in 3 ml of HBSS buffer. A hundred microliters of lung homogenate were used to make the serial dilutions that were then cultured on 7H11 media for CFU analysis. CFU measurements were obtained by averaging two technical replicates each from six biological replicates per strain per timepoint. The CFU limit of detection was 60 CFU/mouse. This study was approved by the ethics committee of the University of Illinois IACUC (protocol ACC # 09–100).

### Macrophage assay

Inhibition of growth of the *Rv0100* KO strain in comparison with its complement strain and WT was assessed as in a macrophage cell culture of J774A.1 cells as previously described [34, 35]. Briefly, J774A.1 cells were grown in DMEM medium supplemented with 10% FBS. Cells were detached and centrifuged, and the pellet was suspended to a final concentration of 1x10^5^ to 3x10^5^ cells/ml. One-milliliter aliquots of cell suspension were distributed into 24-well plates and incubated at 37°C in a 5% CO_2_ incubator for 16h. WT, *Rv0100* KO, and *Rv0100* complement strain cultures were diluted to a final concentration of 4x10^5^ CFU/ml with DMEM, and 500μl of the dilution was dispensed to each well at a 1:1 multiplicity of infection (MOI) and incubated at 37°C for 2 hours to allow for phagocytosis. Cells were washed gently with HBSS to remove the extracellular bacteria, and fresh DMEM medium was added followed by incubation at 37°C under 5% CO_2_. Samples were collected at 0h (for pre-infection controls) and after 1 and 7 days of incubation. Macrophage cells were lysed with 0.25% sodium dodecyl sulfate, and CFU counts were performed by plating on solid agar. The CFU detection limit was 20 CFU/ml. Colonies were counted after 3 weeks of incubation at 37°C. Two biological replicates for CFU counts were prepared for each of three strains at the Pre and day 1 timepoints and 3 biological replicates for the day 7 timepoint. Each biological replicate was plated into two technical replicates for CFU analysis. More biological replicates were planned for the day 7 timepoint in anticipation of potential issues with cell clumping limiting our ability to accurately measure CFUs.

#### RNA isolation and microarray

RNA isolation and microarray were performed as reported previously [36, 37]. Briefly, Cultures of MTB WT H37Rv and the *Rv0100* KO mutant were grown stirring in Dubos-Tween-Albumin (Becton Dickinson) to an OD 600 of 0.15 in vented flasks prior to centrifugation for RNA isolation. RNA isolation was performed by bead agitation in Trizol reagent (Gibco-BRL) to disrupt bacterial cells followed by RNA purification with RNAeasy kits (Qiagen). Labeled cDNA was prepared using Superscript II (Invitrogen) Cy3 or Cy5-dUTP. Labeled cDNA was hybridized overnight to 70-mer oligonucleotide-based microarrays using the tuberculosis oligonucleotide set (Qiagen). Microarrays were scanned using a GenePix 4000A (Axon Instruments). Microarray-determined expression ratios were calculated from three biological replicates of H37Rv versus the *Rv0100* KO and normalized using mean values of the middle 90% of all gene-specific spots on the microarray. Significance analysis of microarrays (SAM) was used to determine statistically significant regulated genes [38]. Only genes regulated at least 2-fold and had a SAM corresponding false discovery q value of zero were included in Table 3, column 1.

**Table 3:** Upregulation of the genes identified in KO *Rv0100* microarray compared to those observed in other dormancy model microarray studies.

Gene	*Rv0100* KO vs WT [This study; S1 Table] n = 3 biological replicates. Values represent mean expression ratio across 2 technical replicates	Upregulated mRNA expr: Starvation under 50% dissolved O_2_, up to 75d [20 (source), 21 (meta-analysis)] Values represent normalized expression ratio [22]	Upregulated mRNA expr: in vitro hypoxia ’Wayne model’, NRP-1 & NRP-2 [8 (source), 21 (meta-analysis)] Values represent normalized expression ratio [22]	Upregulated mRNA expr: in vitro hypoxia ’Wayne model’, NRP-1 & NRP-2 [6 (source), 21 (meta-analysis)] Values represent normalized expression ratio [22]
***Rv0100***	**-**	3.09	-	4.16
***AceA***	5.3	3.5	3.75	-
***AhpC Rv2428***	3.8	2.9	-	2.98
***AhpD Rv2429***	3.2	1.86	3.7	3.065
***RubB***	3.4	1.99	3.49	2.58
***RubA***	3.1	1.45	3.67	2.97
***Rv3249C***	2.8	1.2	3.16	2.42
***Rv3269***	2.5	2.8	-	3.87
***FadE***	2	1.23	3.32	1.56

### Statistical analysis

All statistical comparisons were conducted in Prism (version 9, Graphpad, La Jolla, CA). Log10 transformed CFUs/ml were used as the primary outcome measure in the Wayne, macrophage, and mouse models. Samples were obtained from different strains at various time points and compared using either two-way ANOVA or, in the case of missing data points, a mixed-effects model analysis. Tukey’s test was used for post-hoc follow up testing in the presence of a significant interaction of main effects. It should be noted that cell clumping is an inherent limitation to using CFU as a readout as it makes it difficult to discern individual colonies, particularly with regards to the later time points where cell density is high. To minimize this limitation, all CFU counts were averaged across 2–3 technical replicate plates, as described in detail in the methods section for each experiment. Klett units were used as the primary outcome measure for the qualitative analysis of growth in Sauton’s media experiment. A Klett unit is a measurement of optical density used by a Klett-Summerson Photoelectric Colorimeter (Scienceware, www.belart.com), where 20 Klett units is approximately equal to an OD of 0.1 at 540 nm. This instrument is part of a proprietary system including special culture flasks that allowed us to measure pathogenic anaerobic cultures in a Bio-Safety Level 3 environment.

## Results

### Growth of the Rv0100 KO strain in the Wayne model

**Fig 2:**
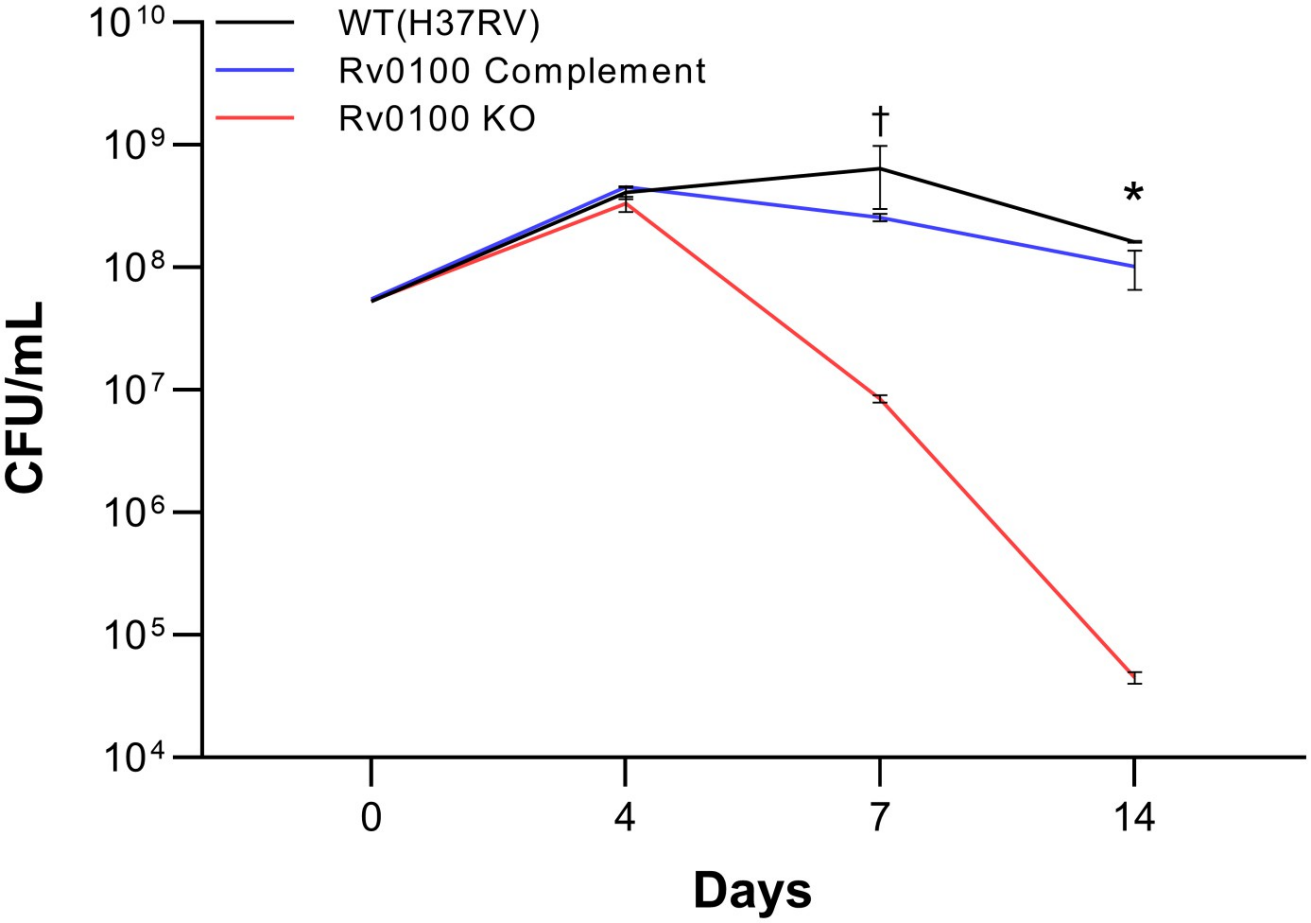
Growth profile of MTB WT, *Rv0100* KO, and its respective complement strain in the Wayne model. The *Rv0100* KO strain showed significantly attenuated growth following exposure to anaerobic conditions for 7 days compared to the complement and WT strains. This effect was further pronounced after 14 days of exposure where the difference in growth reached statistical significance compared to both complement and WT strains. n = 1(day0)-2 biological replicates. Data represented as mean ± SEM CFU/ml across 2 technical replicates per timepoint. Repeated measures mixed-effect ANOVA followed by Tukey’s test. *p<0.05:*Rv0100* KO vs. WT and complement; †p<0.05:*Rv0100* KO vs. complement. CFU = colony forming unit; KO = knock out; WT = wild type.

Given that our hypothesis suggests *Rv0100* could play an essential role during a latent infection, we monitored and compared the growth of our *Rv0100* KO and complement strains to WT in the Non-replicating Persistence model (Wayne model) [30] at several different time points (Fig 2). A mixed model repeated measures analysis uncovered significant main effects of time [F(1.706,3.413) = 142.6, p = 0.0005] and strain [F(2,3) = 122.2, p = 0.0013] as well as a significant time by strain interaction [F(6,6) = 77.08, p<0.0001]. Tukey’s post hoc comparisons test was then used to explore the details of the significant interaction. There was significantly fewer CFUs in the *Rv0100* KO strain compared to the complement strain at day 7 (p = 0.0005) but not the WT strain (p >0.05), likely due to high variability in the samples from this time point. Similarly, there were significantly fewer CFUs in the KO strain at day14 compared to both the WT (p = 0.0170) and the complement strain (p = 0.0281). Overall, these data suggest that knocking out *Rv0100* can lead to significant attenuation of growth following exposure to anaerobic conditions.

### PDIM analysis

**Fig 3:**
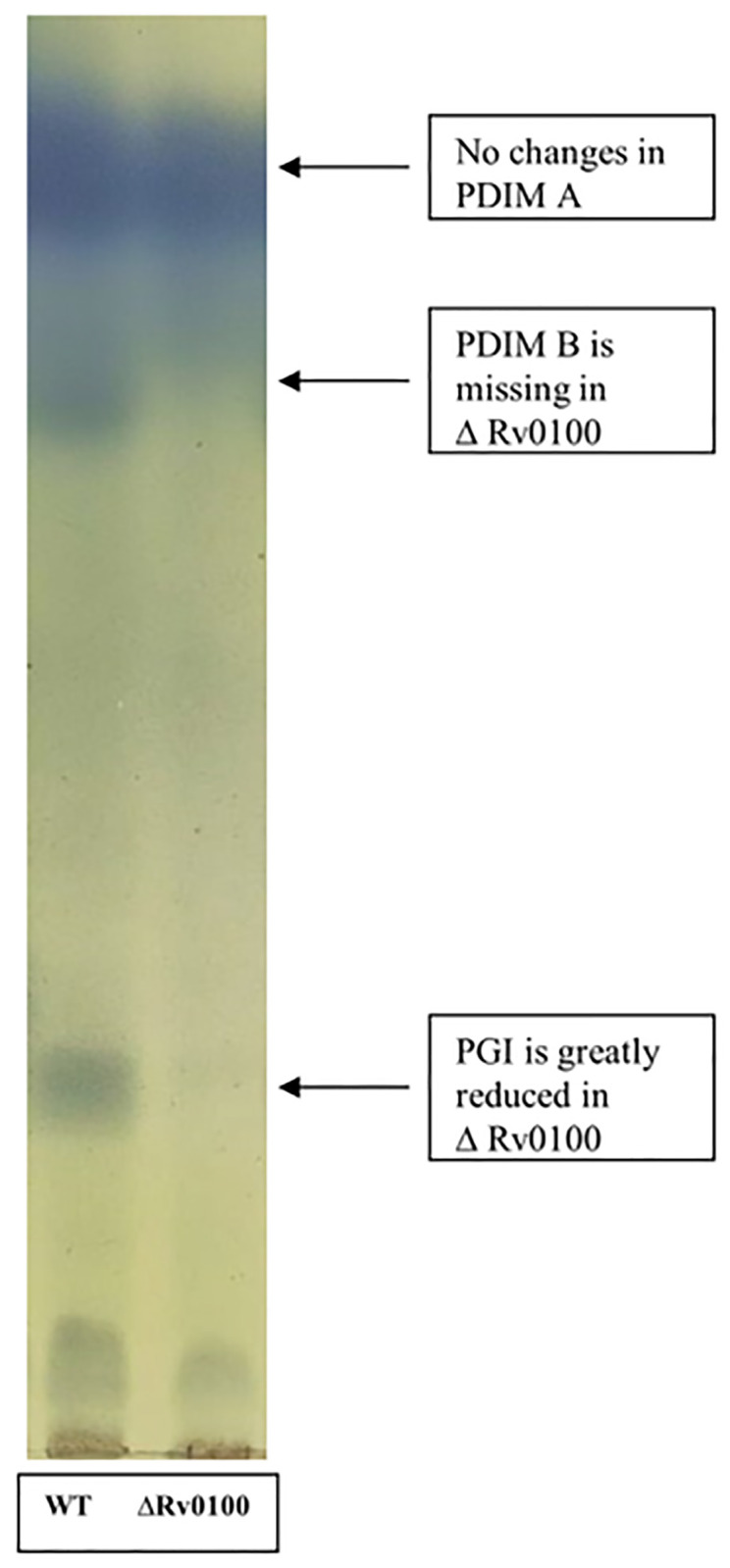
Effect of *Rv0100* KO on PDIM synthesis. Qualitative analysis of lipid extracts from MTB WT and *Rv0100* KO reveals that disruption of the *Rv0100* gene is associated with disrupted synthesis PGI and the virulence enhancing PDIM, DIM B, but not DIM A. In a separate repeat experiment, we also compared the complement strain to WT and found that PGI was restored (S2 Fig). Representative image shown from two biological and two technical replicates. MTB = *Mycobacterium tuberculosis*; WT = wild type; KO = knock out; PDIM = phthiocerol dimycocerosates; PGI = phosphoglucoisomerase: Dim B = dimycocerosates of phthiodiolone; Dim A = dimycocerosates of phthiocerol A.

A qualitative comparison of the bands corresponding to DIM A and DIM B revealed disrupted synthesis of DIM B in the *Rv0100* KO strain but not the WT (Fig 3). PGI was also greatly reduced in the ko strain. No differences in DIM A abundance were observed between the strains. We also examined PDIM content in the *Rv0100* KO complement strain using TLC and found that PGI synthesis was restored (S2 Fig).

### Growth of the Rv0100 KO strain in a mouse model of MTB infection

**Fig 4:**
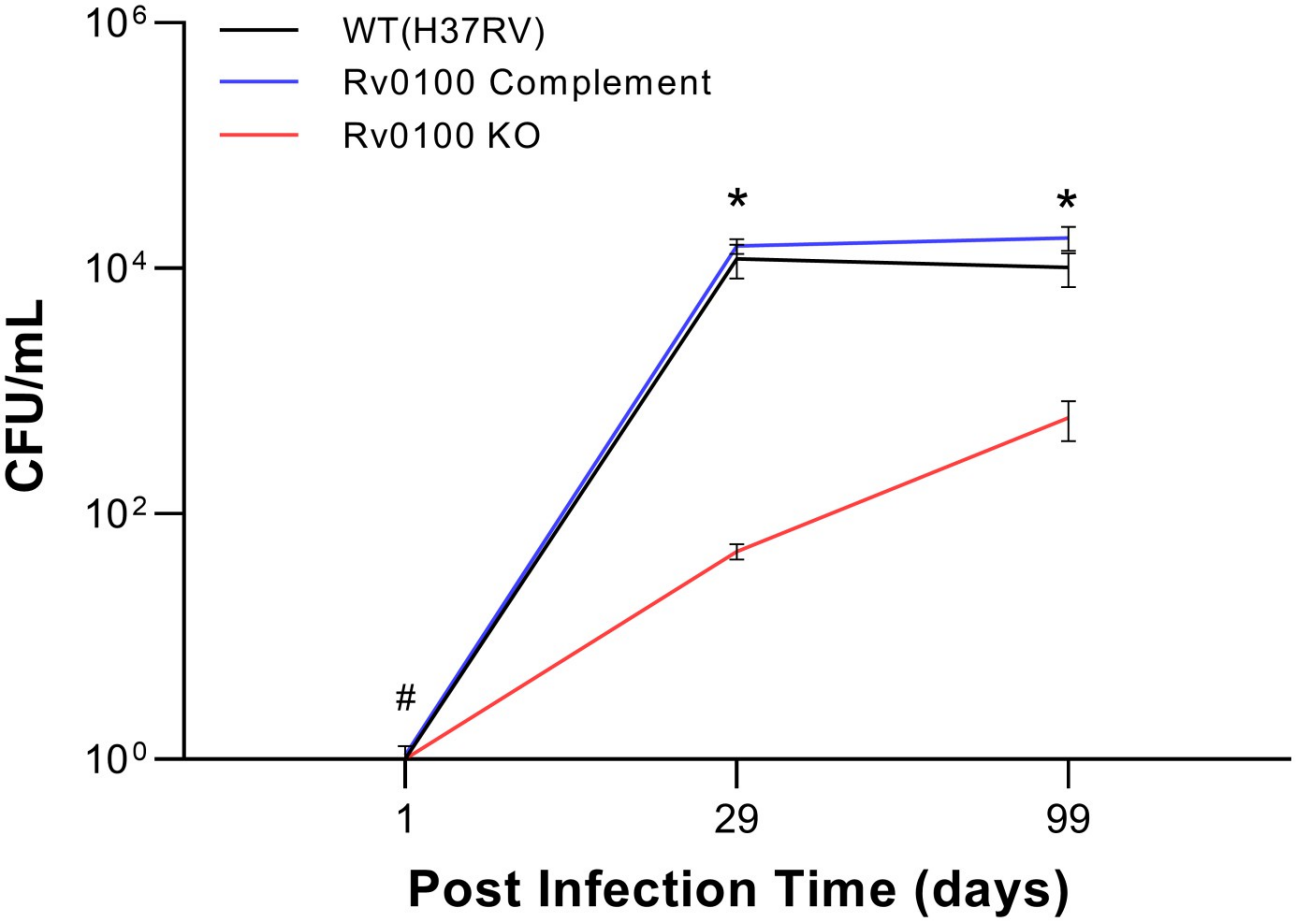
Effect of *Rv0100* KO on growth in the mouse lung. The growth of MTB WT was compared with mutant *Rv0100* KO and complement strains in vivo using a murine aerosol lung infection model. The *Rv0100* KO strain exhibited significantly reduced growth compared to WT at post-infection days 29 and 99. In contrast, restoring *Rv0100* through complementation resulted in a growth profile that was indistinguishable from WT at the same time points. There was also a small, but significant increase in CFUs found in the complement strain versus WT at post-infection day 1. Data represented as mean ± SEM CFU/ml lung tissue homogenate, n = 6 biological replicates per group for each time point. Two-way repeated measures ANOVA followed by Tukey’s test. *p<0.05:*Rv0100* KO vs. WT and complement; #p<0.05:*Rv0100* complement vs. WT. MTB = *Mycobacterium tuberculosis*; CFU = colony forming unit; KO = knock out; WT = wild type.

The effect of *Rv0100* gene knockout on growth in lung tissue compared to both the control WT and the *Rv0100* complement strain was evaluated in vivo using an aerosol infection mouse model (Fig 4). A two-way repeated measures ANOVA revealed significant main effects of both time [F(1.690,25.35) = 791.8, p<0.0001] and strain [F(2,15) = 119.9, p<0.0001] as well as a significant time by strain interaction [F(4,30) = 28.89, p<0.0001]. To further explore this interaction, we used Tukey’s test to compare the CFUs of the KO and complement strains to the control WT strain at each of time points. There were significantly fewer CFUs in the KO strain compared to both WT and complement at day 29 (both p<0.0001) and day 99 (pWT = 0.002, pCOM = 0.0004). In contrast, there were no significant difference between the complement strain and WT at either of these time points (p>0.05). There was also a small, but statistically significant increase in CFUs observed in the complement strain compared to WT at day 1 (mean log10(CFU) ± SD, complement: 0.3095±0.0961; WT: 0.1173±0.0909; p = 0.0132), however the cause of this difference is not clear. Together, these results indicate that knocking out *Rv0100* attenuates the growth of this mutant MTB strain compared to WT MTB in the mouse lung and that restoring the *Rv0100* gene through complementation can rescue this deficit.

### Growth of the Rv0100 KO strain in a macrophage model

**Fig 5:**
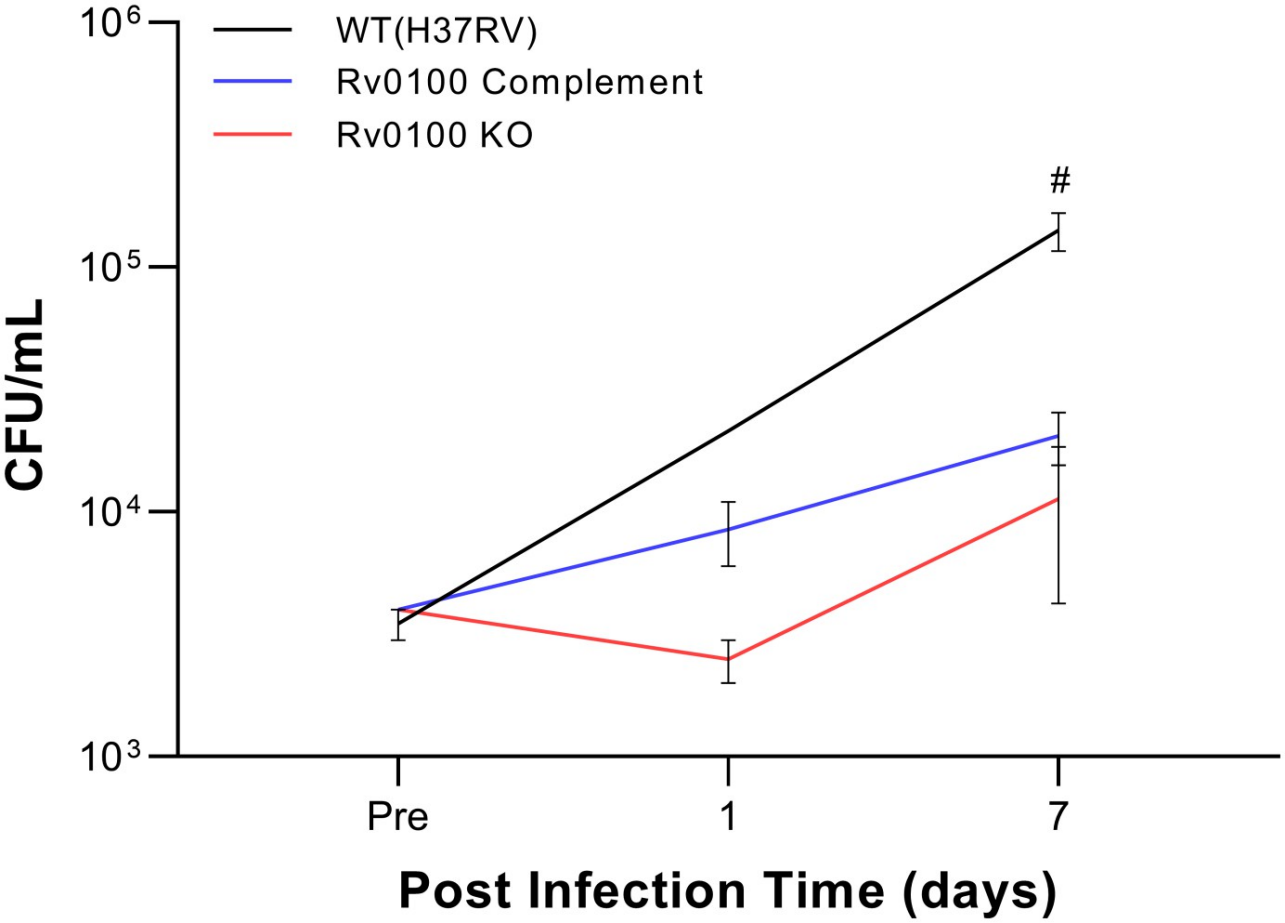
Effect of *Rv0100* KO on intracellular survivability in J774A macrophages. The ability of the mutant *Rv0100* KO strain to survive phagocytosis by macrophages was assessed by comparing its growth with MTB WT and the *Rv0100* complement strains at three time points. The results showed a significant reduction of CFUs in *Rv0100* KO strain compared to complement at post-infection day 7. The WT strain also exhibited attenuation at this time point but the effect did not reach statistical significance. n = 1(day1;WT)-3 biological replicates per strain for each time point. Data represented as mean ± SEM CFU/ml across n = 1(day1;WT)-3 technical replicates per group for each time point. Repeated measures mixed-effect ANOVA followed by Tukey’s test. #p<0.05:*Rv0100* complement vs. WT. MTB = *Mycobacterium tuberculosis*; CFU = colony forming unit; KO = knock out; WT = wild type.

We next sought to determine the effects of *Rv0100* KO on the survivability of MTB in J774A.1 macrophages (Fig 5). Macrophages were infected with 4x10^5^ CFU/ml of either WT, *Rv0100* KO, or the *Rv0100* complement strain and allowed to incubate for 0h (Pre), 1 day or 7 days before collecting the lysate and culturing the surviving MTB from each condition. All cultures were visually inspected under the microscope before lysis at each time point. No differences between the MTB strain conditions were noted neither in appearance following the initial phagocytosis, nor in macrophage viability. A mixed effects ANOVA of the resulting CFUs found a significant main effect of time [F(1.275,3.187) = 87.38, p = 0.0019] and a significant time by strain interaction [F(4,5) = 31.65, p = 0.0010]. A follow up Tukey’s test revealed a significant attenuation of growth in the *Rv0100* complement strain compared to WT (p = 0.0083) at the 7 day post-infection time point. While the growth of the *Rv0100* KO strain also appeared attenuated compared to WT at this time point, the effect did not reach statistical significance (p = 0.0636). It should be noted that there were two biological replicates planned for each of three strains at the Pre and day 1 timepoints and 3 biological replicates for the day 7 timepoint. However, one of the two biological replicates for WT failed at the day 1 timepoint and was not measurable. Overall, these data suggest that *Rv0100* may play an important role in the survivability of MTB after phagocytosis by macrophages, however due to high statistical variability, this conclusion is limited to being based on qualitative observations of the differences in growth profile between the strains.

### Global gene expression profiling of the Rv0100 KO strain

To determine the regulatory scope of *Rv0100*, we performed global gene expression profiling using RNA microarrays comparing the *Rv0100* KO strain with the H37Rv WT strain (S1 Table). While comparing *Rv0100* KO vs. WT, we found several genes to be significantly upregulated using the SAM statistical analysis technique [38]. Amongst them, *Rv0467* (*aceA*, isocitrate lyase) showed the most significant difference at 5.3-fold. Other notable genes displayed modest induction: *AhpC* (Alkyl hydroperoxidase), 3.8-fold; *AhpD* (member of AhpC/TSA family), 3.2-fold; *RubA* (rubredoxin A), 3.4-fold; *RubB* (rubredoxin B), 3.1-fold; *Rv3249C* (transcriptional regulator, TetR/AcrR family), 2.8-fold; *Rv3269* (probable heat shock protein), 2.5-fold; and *FadE23* (acyl coA dehydrogenase), 2-fold. In contrast, we also found several down regulated genes including *Rv1361c* (member of PPE family), 10.7-fold; *LipF* (a probable esterase), 3.5-fold; *Rv0171* (part of the mce operon) 3.3-fold; *Rv0173* (part of mce operon), 2.7-fold; *Rv2295*, 2.5-fold; *Rv3723*, 2.5-fold; and *Rv2840C*, 2-fold. We then compared our list of up-regulated genes with normalized expression data of other microarray studies evaluated in a large-scale meta-analysis [22]. We focused our comparisons on the expression pattern changes found in WT following exposure to hypoxic conditions for genes regulated at least 2-fold with a SAM corresponding false discovery q value of zero (Table 3). We found that knocking out *Rv0100* produced significant changes in gene expression patterns that partially overlap patterns exhibited by WT strains under hypoxic conditions. Overall, these results support the role of *Rv0100* as a potential mediator of the dormancy phenotype exhibited by MTB under hypoxic conditions.

## Discussion

MTB has a unique ability to enter a state of dormancy during latent TB infection. The dormancy phenotype is a critical aspect of the bacterium’s life cycle and has significant implications for the spread and treatment of TB. During latent infection, MTB can persist in a non-replicating state within the host for years or even decades without causing symptoms. This state of dormancy allows the bacterium to evade the host’s immune response and survive in an environment that is hostile to its growth and replication. The immune system of the host keeps the bacteria under control but cannot eradicate it completely, leading to a delicate balance between the host and the pathogen. In this study have demonstrated that *Rv0100* is not only essential for MTB growth in the Wayne model of non-replicating persistence, but also crucial for the pathogen’s survival in a macrophage assay as well as in a mouse model of TB infection.

A key challenge in tuberculosis research is interpreting genomic data within the context of host-pathogen interactions and leveraging this knowledge to develop a new generation of drugs and vaccines. The complete genome sequences of the MTB pathogen and the human host have been available since 2001, thanks to The Genome International Consortium. Analyzing these genomes enables us to identify potential gene products unique to the pathogen and absent in the host. This significantly narrows down the search for viable drug targets from a vast array to a select few. Most known antibacterial compounds function by inhibiting specific bacterial enzymes or proteins. Enzymes in MTB pathways that are dissimilar to any human protein are particularly promising as drug targets. In this study, we focused on the ACP, Rv0100, which is one of several proteins that play a role in the bacterial fatty acid biosynthesis pathway (FasII pathway) of MTB [39, 40], in contrast to the human FasI pathway, which utilizes a single multi-enzyme complex. Since the MTB FasII pathway is not similar to its human counterpart, *Rv0100* represents a potentially attractive drug target as it would be likely to have minimal off-target effects.

Fatty acid metabolism is one of the primary targets for first-line antibacterial therapeutics. One ubiquitous example of a drug that targets bacterial fatty acid synthesis is the widely used Fabl inhibitor, Triclosan, which is usually directed against pathogenic *Staphylococcus* and *E*. *coli* infections. Fatty acid precursors are likely important for energy storage and cell wall synthesis that is required for maintenance of bacterial dormancy. In particular, MTB uses triacylglycerol to accumulate lipid droplets to acquire a dormancy-like phenotype [11]. Among the first-line therapeutics against MTB, isoniazid stands out for its targeted action on FasII system, crucial for the synthesis of mycolic acids, key components of the mycobacterial cell wall. This mechanism underscores the drug’s efficacy in disrupting the bacterial cell envelope, a vital barrier and structural component of MTB. Furthermore, the seminal report by Cole et. al (1998) predicted several MTB ACPs that would likely be required for the synthesis of mycolic acids [19]. These additional ACPs include Rv2244, Rv0033, and Rv1344. However, while these proteins share an identity of 15–23% and a similarity of 26–39% with Rv0100, our microarray analysis and comparisons with other dormancy models found no changes in the expression pattern of these ACPs. These results support our previous hypothesis that Rv0100 has more potential as a drug target than these other ACPs. Finally, several drugs are entering phase II trials to inhibit cell wall synthesis in MTB [41]. Taken together, these developments are highly suggestive of the promise held by inhibitors of fatty acid metabolism in combating MTB.

As previously mentioned, FADDs and FAAL are essential for linking fatty acids and polyketide synthesis in Mycobacterium [12, 13] and FAAL is responsible for transferring fatty acids to Rv0100 [14]. Additionally, there are multiple acyl-AMP ligases that are required for biosynthesis of PDIMs, which are important virulence lipids in the cell wall [42–45]. Interestingly, the disruption of PDIM synthesis observed in the *Rv0100* KO strain in our study is similar to those found in *M*. *bovis* by Hotter et al. (2005) following the mutation of *MB0100* (*Rv0097* equivalent). Together, these findings may be indicative of a protein partner relationship between Rv0100 and Rv0097. Another study found that disruption of the ppe1-nrp operon harboring *Rv0100* resulted in a disruption of PDIM synthesis but found no direct link to *Rv0100* itself [46]. While outside the scope of the current study, it will be important for future work to investigate the specific role of *Rv0100* in this context in order to further clarify the mechanisms of PDIM synthesis and its impact on virulence.

A comprehensive meta-analysis has shown that, across multiple sets of data, there is upregulation of *Rv0100* in models of MTB dormancy [22]. This study normalizes experimental data gathered from microarray DNA gene expression ratios, accounting for maximum time end point from the source materials. Here, we compared our microarray results with the source data sets from the meta-analysis that used hypoxic conditions [6, 8, 21] and found a substantial overlap of genes, including *Rv0100*, that are upregulated (Table 3) [22]. In the microarray studies with starvation under 50% dissolved O_2_ [21], and microarray studies mimicking the Wayne model [6], *Rv0100* was upregulated 3-fold and 4-fold, respectively. However, no changes were seen for *Rv0100* in one of the comparative studies [8]. Others have also shown that the expression of *Rv0100* was induced 2.2-fold by diamide, a chemical which triggers an oxidative stress reaction [47].

Collectively, the results from our work as well as others strongly implicate that *Rv0100* is upregulated in response to oxidative stress and likely plays a key role in its survival during latent infections, potentially by mediating its dormancy state. However, future work needs to be performed to expand the scope of the current study in way that can firmly establish the essentiality of *Rv0100* in the dormancy state of MTB and any potential role it may have on the synthesis of PDIMs. Only through increasing our understanding of how MTB survives during latent infection can we effectively develop desperately needed drugs to combat this ancient and deadly bacterium.

## Supporting information

S1 Fig Growth profile of MTB WT and *Rv0100* KO in Sauton’s media in the presence of 2% or 6% glycerol. The *Rv0100* KO strain showed severely attenuated growth in the presence of 6% glycerol. Growth was achieved in the presence of 2% glycerol; however, it was much slower than WT in comparable media. n = 1 technical replicate per condition. KO = knock out; WT = wild type.
(TIF)

S2 Fig Lipid analysis of MTB WT versus the Rv0100 KO strain and the complement strain. The various strains of MTB were grown in Sauton’s medium and bacterial cells were harvested in chloroform/methanol and lipids were subjected to thin layer chromatography. After resolving lipids on a thin layer plate, they were scanned using the ultra-violet scanner to visualize the bands. n = 3 biological replicates. Representative figure shown from three technical replicates. The blue arrows indicate PDI. The numbers represent Wild type (WT), *Rv0100* KO (220) and Complement (227).
(TIF)

S1 Table Global gene expression profiling. We performed global gene expression profiling using RNA microarrays to compare the *Rv0100* KO strain with WT.
(PDF)

S1 Raw images Original image of Fig 3 TLC gel. (PDF)
