# Supplementary figures and images for: Rv0100: An essential acyl carrier protein from M. tuberculosis important in dormancy
Source: PLoS One. 2024 Jun 7;19(6):e0304876. doi: 10.1371/journal.pone.0304876 (PMC11161019; doi:10.1371/journal.pone.0304876)

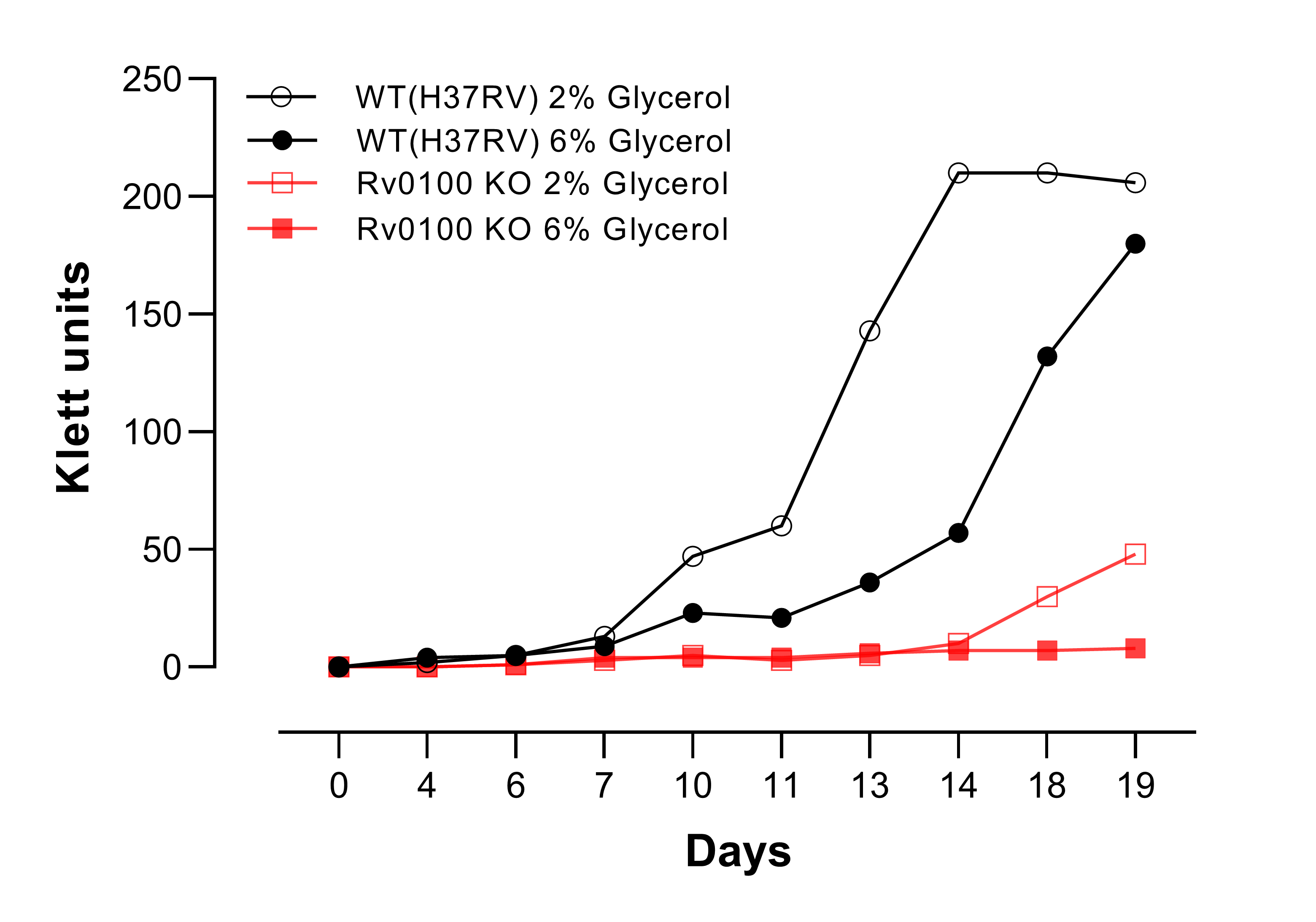

Supplement: S1 Fig — The Rv0100 KO strain showed severely attenuated growth in the presence of 6% glycerol. Growth was achieved in the presence of 2% glycerol; however, it was much slower than WT in comparable media. n = 1 technical replicate per condition. KO = knock out; WT = wild type. (TIF) [file pone.0304876.s001.tif]

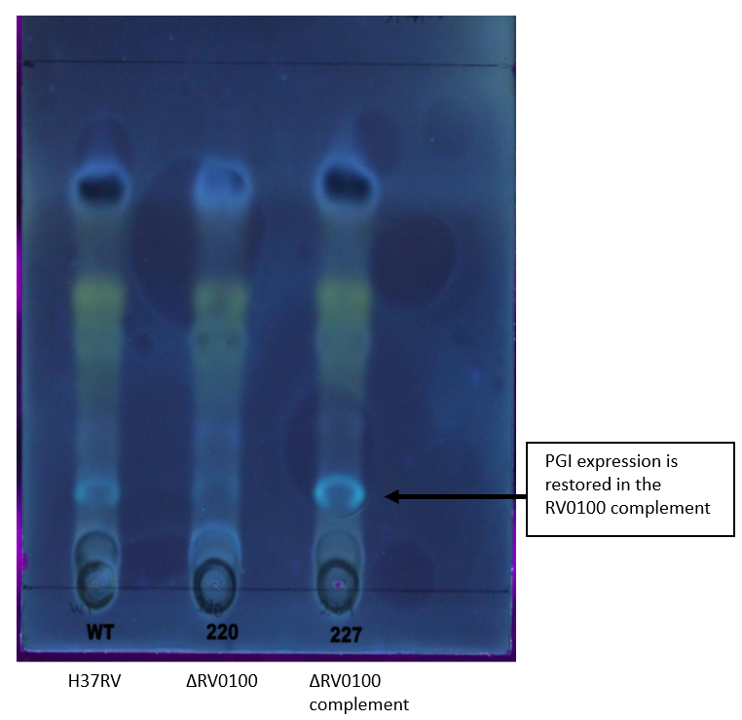

Supplement: S2 Fig — The various strains of MTB were grown in Sauton’s medium and bacterial cells were harvested in chloroform/methanol and lipids were subjected to thin layer chromatography. After resolving lipids on a thin layer plate, they were scanned using the ultra-violet scanner to visualize the bands. n = 3 biological replicates. Representative figure shown from three technical replicates. The blue arrows indicate PDI. The numbers represent Wild type (WT), Rv0100 KO (220) and Complement (227). (TIF) [file pone.0304876.s002.tif]

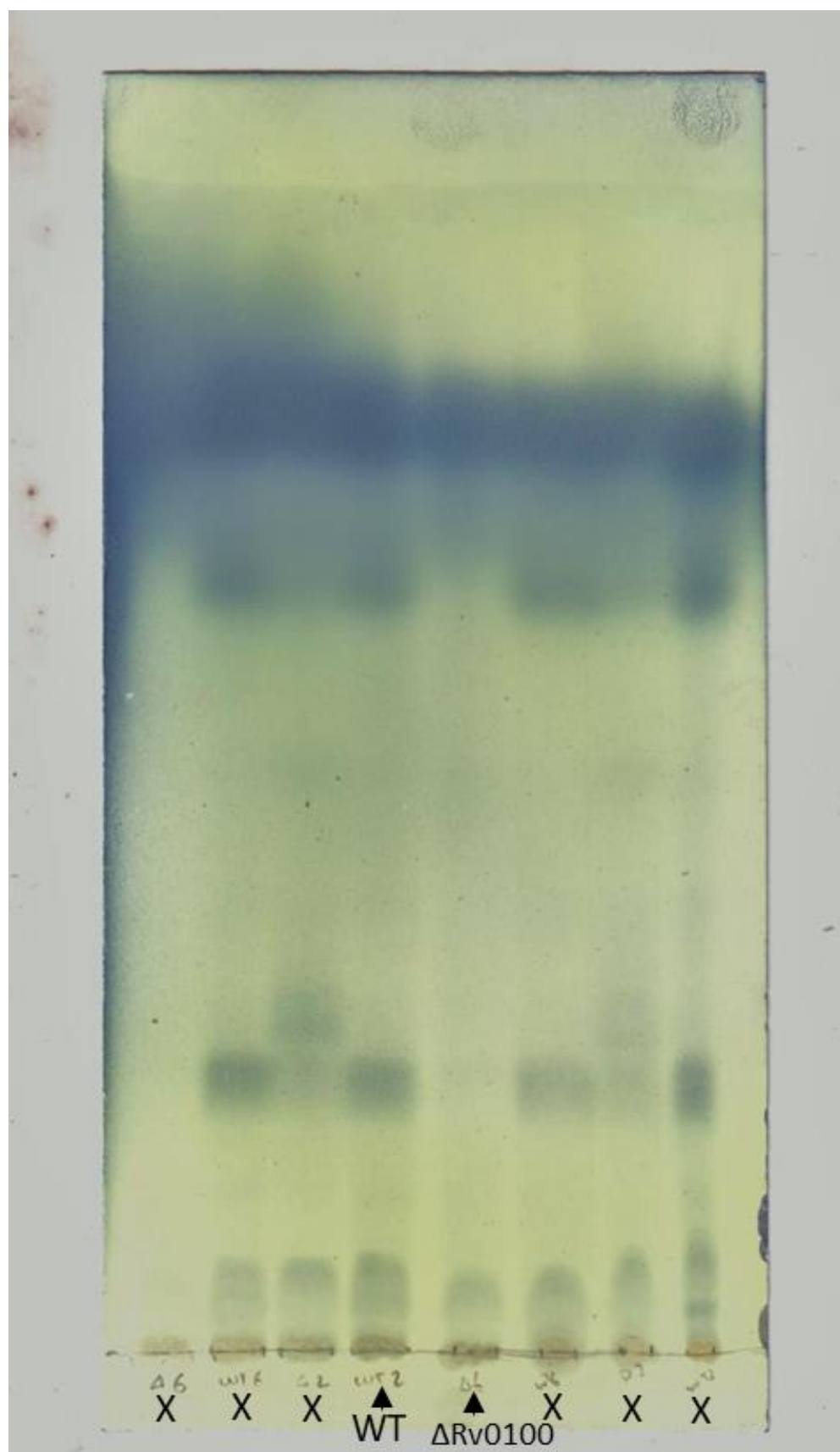

Original Image from TLC represented in Fig 3 from main manuscript

Supplement: S1 Raw images — (PDF) [file pone.0304876.s004.pdf]
