# Supplementary material for: Rv0100: An essential acyl carrier protein from M. tuberculosis important in dormancy
Source: PLoS One. 2024 Jun 7;19(6):e0304876. doi: 10.1371/journal.pone.0304876 (PMC11161019; doi:10.1371/journal.pone.0304876)
Supplement: S1 Table — We performed global gene expression profiling using RNA microarrays to compare the Rv0100 KO strain with WT. (PDF) [file pone.0304876.s003.pdf]

TVB304 upreg.

Rv	Gene	Corrected intensity Average CH1 Cye5	CH2 Cye3	Average Corrected ratios CH2/CH1 Induced	Average Corrected ratios CH1/CH2 Repressed	Gene product
Rv0287	*Rv0287*	1195	621	1.9	0.5	conserved hypothetical protein
Rv0350	*dnaK*	616	331	1.9	0.5	70 kD heat shock protein, chromosome replication
Rv0389	*purT*	315	10	31.0	0.0	phosphoribosylglycinamide formyltransferase II
Rv0467	*aceA*	223	36	6.3	0.2	isocitrate lyase
Rv0925c	*Rv0925c*	212	111	1.9	0.5	hypothetical protein
Rv1343c	*Rv1343c*	79	38	2.1	0.5	conserved hypothetical protein
Rv1592c	*Rv1592c*	205	105	2.0	0.5	conserved hypothetical protein
Rv1653	*argJ*	109	51	2.1	0.5	glutamate N-acetyltransferase
Rv2002	*fabG3*	89	38	2.4	0.4	3-oxoacyl-[ACP] reductase
Rv2284	*lipM*	260	121	2.2	0.5	probable esterase
Rv2428	*ahpC*	428	132	3.2	0.3	alkyl hydroperoxide reductase
Rv2429	*ahpD*	465	147	3.2	0.3	member of AhpC/TSA family
Rv2741	*PE_PGRS*	82	18	4.5	0.2	PE_PGRS-family protein
Rv2822c	*Rv2822c*	82	37	2.2	0.4	hypothetical protein
Rv2868c	*gcpE*	161	36	4.5	0.2	essential gene of unknown function
Rv2980	*Rv2980*	85	28	3.0	0.3	hypothetical protein
Rv3020c	*PE*	458	246	1.9	0.5	PE-family protein
Rv3074	*Rv3074*	1141	123	9.3	0.1	conserved hypothetical protein
Rv3140	*fadE23*	345	182	1.9	0.5	acyl-CoA dehydrogenase
Rv3249c	*Rv3249c*	97	41	2.4	0.4	transcriptional regulator (TetR/AcrR family)
Rv3250c	*rubB*	253	98	2.6	0.4	rubredoxin B
Rv3251c	*rubA*	246	88	2.8	0.4	rubredoxin A
Rv3269	*Rv3269*	198	102	1.9	0.5	probable heat shock protein
Rv3301c	*phoY1*	581	205	2.8	0.4	phosphate transport system regulator
Rv3432c	*gadB*	99	13	7.5	0.1	glutamate decarboxylase
Rv3478	*PPE*	1200	562	2.1	0.5	PPE-family protein
Rv3481c	*Rv3481c*	335	164	2.0	0.5	possible membrane protein

TVB305 upreg.

		Corrected intensity		Average	Average	
		Average		Corrected rati	rrected ratios	
Rv	Gene	CH1	CH2	CH2/CH1	CH1/CH2	ene product
		Cye5	Cye3	Induced	Repressed	
Rv0128	*Rv0128*	217	51	4.3	0.2	hypothetical protein
Rv0235c	*Rv0235c*	189	14	13.5	0.1	conserved hypothetical protein
Rv0467	*aceA*	241	56	4.3	0.2	isocitrate lyase
Rv0905	*echA6*	191	89	2.1	0.5	enoyl-CoA hydratase/isomerase superfamily
Rv1130	*Rv1130*	144	29	5.0	0.2	conserved hypothetical protein
Rv1131	*gltA1*	121	23	5.4	0.2	citrate synthase 3
Rv1221	*sigE*	461	234	2.0	0.5	ECF subfamily sigma subunit
Rv1528c	*papA4*	627	1281	0.5	2.0	PKS-associated protein, unknown function
Rv1668c	*Rv1668c*	188	9	21.8	0.0	probable ABC transporter
Rv1736c	*narX*	94	4	21.8	0.0	fused nitrate reductase
Rv1779c	*Rv1779c*	174	81	2.2	0.5	possible integral membrane protein
Rv2255c	*Rv2255c*	193	15	12.8	0.1	hypothetical protein
Rv2428	*ahpC*	196	45	4.3	0.2	alkyl hydroperoxide reductase
Rv2429	*ahpD*	490	152	3.2	0.3	member of AhpC/TSA family
Rv2624c	*Rv2624c*	128	41	3.1	0.3	conserved hypothetical protein
Rv2842c	*Rv2842c*	131	34	3.8	0.3	conserved hypothetical protein
Rv3094c	*Rv3094c*	260	89	2.9	0.3	conserved hypothetical protein
Rv3140	*fadE23*	331	158	2.1	0.5	acyl-CoA dehydrogenase
Rv3210c	*Rv3210c*	138	53	2.6	0.4	hypothetical protein
Rv3249c	*Rv3249c*	106	32	3.3	0.3	transcriptional regulator (TetR/AcrR family)
Rv3250c	*rubB*	214	59	3.6	0.3	rubredoxin B
Rv3251c	*rubA*	281	70	4.0	0.2	rubredoxin A
Rv3269	*Rv3269*	323	101	3.2	0.3	probable heat shock protein
Rv3804c	*fbpA*	3517	1796	2.0	0.5	antigen 85A, mycolyltransferase
Rv3837c	*Rv3837c*	285	25	11.5	0.1	putative phosphoglycerate mutase

TVB304 downreg.

Rv	Gene	Corrected intensity Average CH1 Cye5	CH2 Cye3	Average Corrected ratios CH2/CH1 Induced	Average Corrected ratios CH1/CH2 Repressed	Gene product
Rv0001	*dnaA*	74	176	0.4	2.4	chromosomal replication initiator protein
Rv0011c	*Rv0011c*	48	112	0.4	2.3	hypothetical protein
Rv0060	*Rv0060*	53	112	0.5	2.1	hypothetical protein
Rv0167	*Rv0167*	57	123	0.5	2.2	part of mce1 operon
Rv0169	*mce1*	144	349	0.4	2.4	cell invasion protein
Rv0171	*Rv0171*	68	193	0.4	2.8	part of mce1 operon
Rv0173	*lprK*	109	313	0.3	2.9	part of mce1 operon
Rv0174	*Rv0174*	87	175	0.5	2.0	part of mce1 operon
Rv0179c	*lprO*	87	177	0.5	2.0	lipoprotein
Rv0207c	*Rv0207c*	66	132	0.5	2.0	conserved hypothetical protein
Rv0250c	*Rv0250c*	103	234	0.4	2.3	hypothetical protein
Rv0341	*Rv0341*	272	625	0.4	2.3	conserved hypothetical protein
Rv0342	*Rv0342*	42	111	0.4	2.6	conserved hypothetical protein
Rv0364	*Rv0364*	69	181	0.4	2.6	conserved hypothetical protein
Rv0513	*Rv0513*	67	141	0.5	2.1	probable membrane protein
Rv0655	*Rv0655*	154	402	0.4	2.6	ABC transporter
Rv0724	*sppA*	64	130	0.5	2.0	protease IV, signal peptide peptidase
Rv1107c	*xseB*	49	111	0.4	2.3	exonuclease VII small subunit
Rv1115	*Rv1115*	66	153	0.4	2.3	hypothetical protein
Rv1131	*gltA1*	55	132	0.4	2.4	citrate synthase 3
Rv1166	*lpqW*	52	146	0.4	2.8	lipoprotein
Rv1216c	*Rv1216c*	121	170	0.7	1.4	conserved hypothetical protein
Rv1240	*mdh*	360	289	1.2	0.8	malate dehydrogenase
Rv1361c	*PPE*	69	536	0.1	7.8	PPE-family protein
Rv1396c	*PE_PGRS*	65	147	0.4	2.3	PE_PGRS-family protein
Rv1397c	*Rv1397c*	220	661	0.3	3.0	conserved hypothetical protein
Rv1398c	*Rv1398c*	158	330	0.5	2.1	conserved hypothetical protein
Rv1458c	*Rv1458c*	58	145	0.4	2.5	ABC-type transporter
Rv1460	*Rv1460*	148	151	1.0	1.0	putative transcriptional regulator
Rv1462	*Rv1462*	93	110	0.8	1.2	conserved hypothetical protein
Rv1484	*inhA*	255	193	1.3	0.8	enoyl-[ACP] reductase
Rv1500	*Rv1500*	50	113	0.4	2.3	similarity to B. subtilis glycosyltransferase
Rv1530	*adh*	105	121	0.9	1.2	alcohol dehydrogenase (Zn)
Rv1612	*trpB*	35	116	0.3	3.3	tryptophan synthase [beta] chain
Rv1614	*lgt*	87	203	0.4	2.3	prolipoprotein diacylglyceryl transferase
Rv1709	*Rv1709*	64	147	0.4	2.3	conserved hypothetical protein
Rv1870c	*Rv1870c*	158	378	0.4	2.4	hypothetical protein
Rv1906c	*Rv1906c*	103	285	0.4	2.8	conserved hypothetical protein
Rv2097c	*Rv2097c*	69	137	0.5	2.0	conserved hypothetical protein
Rv2166c	*Rv2166c*	58	137	0.4	2.4	conserved hypothetical protein
Rv2295	*Rv2295*	102	274	0.4	2.7	conserved hypothetical protein
Rv2444c	*rne*	85	187	0.5	2.2	similar at C-term to ribonuclease E
Rv2475c	*Rv2475c*	72	148	0.5	2.1	conserved hypothetical protein
Rv2499c	*Rv2499c*	52	127	0.4	2.4	putative aldehyde dehydrogenase
Rv2657c	*Rv2657c*	135	271	0.5	2.0	similar to gp36 of mycobacteriophage L5
Rv2785c	*rpsO*	70	173	0.4	2.5	30S ribosomal protein S15
Rv2840c	*Rv2840c*	562	1125	0.5	2.0	conserved hypothetical protein
Rv2929	*Rv2929*	120	239	0.5	2.0	hypothetical protein
Rv2930	*fadD26*	81	171	0.5	2.1	acyl-CoA synthase
Rv2932	*ppsB*	76	153	0.5	2.0	phenolpthiocerol synthesis (pksC)
Rv2950c	*fadD29*	102	218	0.5	2.1	acyl-CoA synthase
Rv2970c	*lipN*	936	2488	0.4	2.7	probable lipase/esterase
Rv2988c	*leuC*	55	118	0.5	2.1	3-isopropylmalate dehydratase large subunit
Rv3058c	*Rv3058c*	53	126	0.4	2.4	putative transcriptional regulator
Rv3146	*nuoB*	59	134	0.4	2.3	NADH dehydrogenase chain B
Rv3150	*nuoF*	139	279	0.5	2.0	NADH dehydrogenase chain F
Rv3152	*nuoH*	62	164	0.4	2.6	NADH dehydrogenase chain H
Rv3154	*nuoJ*	67	143	0.5	2.1	NADH dehydrogenase chain J
Rv3156	*nuoL*	94	188	0.5	2.0	NADH dehydrogenase chain L
Rv3295	*Rv3295*	85	175	0.5	2.1	transcriptional regulator (TetR/AcrR family)
Rv3339c	*icd1*	42	116	0.4	2.8	isocitrate dehydrogenase
Rv3464	*rmlB*	136	276	0.5	2.0	dTDP-glucose 4,6-dehydratase
Rv3487c	*lipF*	43	158	0.3	3.7	probable esterase
Rv3490	*otsA*	63	137	0.5	2.2	probable [alpha],-trehalose-phosphate synthase
Rv3680	*Rv3680*	116	238	0.5	2.0	probable anion transporter
Rv3723	*Rv3723*	48	130	0.4	2.7	hypothetical protein
Rv3822	*Rv3822*	176	406	0.4	2.3	conserved hypothetical protein
Rv3872	*PE*	52	109	0.5	2.1	PE-family protein
Rv3920c	*Rv3920c*	213	425	0.5	2.0	conserved hypothetical protein
Rv3923c	*rnpA*	76	156	0.5	2.1	ribonuclease P protein component
Rv3924c	*rpmH*	160	338	0.5	2.1	50S ribosomal protein L34
Rv3924c	*rpmH*	167	347	0.5	2.1	50S ribosomal protein L34

TVB305 downreg.

Rv	Gene	Corrected intensity Average CH1 Cye5	CH2 Cye3	Average corrected ratios CH2/CH1 Induced	Average corrected ratios CH1/CH2 Repressed	Gene product
Rv0003	*recF*	50	95	0.5	1.9	DNA replication and SOS induction
Rv0047c	*Rv0047c*	44	98	0.4	2.2	conserved hypothetical protein
Rv0167	*Rv0167*	83	244	0.3	2.9	part of mce1 operon
Rv0169	*mce1*	112	387	0.3	3.5	cell invasion protein
Rv0171	*Rv0171*	49	188	0.3	3.8	part of mce1 operon
Rv0172	*Rv0172*	36	93	0.4	2.6	part of mce1 operon
Rv0173	*lprK*	148	369	0.4	2.5	part of mce1 operon
Rv0179c	*lprO*	67	131	0.5	2.0	lipoprotein
Rv0297	*PE_PGRS*	71	132	0.5	1.9	PE_PGRS-family protein
Rv0341	*Rv0341*	471	1068	0.4	2.3	conserved hypothetical protein
Rv0364	*Rv0364*	56	107	0.5	1.9	conserved hypothetical protein
Rv0655	*Rv0655*	227	476	0.5	2.1	ABC transporter
Rv1115	*Rv1115*	95	188	0.5	2.0	hypothetical protein
Rv1346	*fadE14*	92	172	0.5	1.9	acyl-CoA dehydrogenase
Rv1361c	*PPE*	46	625	0.1	13.6	PPE-family protein
Rv1397c	*Rv1397c*	297	553	0.5	1.9	conserved hypothetical protein
Rv1517	*Rv1517*	53	131	0.4	2.5	conserved hypothetical protein
Rv1528c	*papA4*	627	1281	0.5	2.0	PKS-associated protein, unknown function
Rv1597	*Rv1597*	46	93	0.5	2.0	hypothetical protein
Rv1639c	*Rv1639c*	70	137	0.5	2.0	conserved hypothetical protein
Rv1641	*infC*	315	715	0.4	2.3	initiation factor IF-3
Rv1871c	*Rv1871c*	336	642	0.5	1.9	hypothetical protein
Rv1887	*Rv1887*	74	143	0.5	1.9	hypothetical protein
Rv1904	*Rv1904*	65	126	0.5	1.9	conserved hypothetical protein
Rv1906c	*Rv1906c*	99	186	0.5	1.9	conserved hypothetical protein
Rv2138	*lppL*	58	133	0.4	2.3	lipoprotein
Rv2190c	*Rv2190c*	64	136	0.5	2.1	putative p60 homologue
Rv2295	*Rv2295*	86	185	0.5	2.2	conserved hypothetical protein
Rv2331	*Rv2331*	49	98	0.5	2.0	hypothetical protein
Rv2391	*nirA*	92	172	0.5	1.9	probable nitrite reductase/sulphite reductase
Rv2753c	*dapA*	43	94	0.5	2.2	dihydrodipicolinate synthase
Rv2837c	*Rv2837c*	50	94	0.5	1.9	conserved hypothetical protein
Rv2840c	*Rv2840c*	504	1031	0.5	2.0	conserved hypothetical protein
Rv2846c	*efpA*	78	157	0.5	2.0	putative efflux protein
Rv3145	*nuoA*	67	142	0.5	2.1	NADH dehydrogenase chain A
Rv3146	*nuoB*	45	94	0.5	2.1	NADH dehydrogenase chain B
Rv3150	*nuoF*	116	225	0.5	1.9	NADH dehydrogenase chain F
Rv3154	*nuoJ*	68	127	0.5	1.9	NADH dehydrogenase chain J
Rv3156	*nuoL*	44	140	0.3	3.2	NADH dehydrogenase chain L
Rv3487c	*lipF*	74	237	0.3	3.2	probable esterase
Rv3614c	*Rv3614c*	63	130	0.5	2.1	conserved hypothetical protein
Rv3616c	*Rv3616c*	85	211	0.4	2.5	conserved hypothetical protein
Rv3680	*Rv3680*	71	132	0.5	1.9	probable anion transporter
Rv3723	*Rv3723*	67	161	0.4	2.4	hypothetical protein
